# Immune cell proportions correlate with clinicogenomic features and *ex vivo* drug responses in acute myeloid leukemia

**DOI:** 10.3389/fonc.2023.1192829

**Published:** 2023-06-08

**Authors:** Kyle A. Romine, Daniel Bottomly, William Yashar, Nicola Long, Matthew Viehdorfer, Shannon K. McWeeney, Jeffrey W. Tyner

**Affiliations:** ^1^ Knight Cancer Institute, Oregon Health & Science University, Portland, OR, United States; ^2^ Division of Bioinformatics and Computational Biology, Department of Medical Informatics and Clinical Epidemiology, Oregon Health & Science University, Portland, OR, United States; ^3^ School of Medicine, Oregon Health & Science University, Portland, OR, United States; ^4^ Division of Hematology & Medical Oncology, Department of Medicine, Oregon Health & Science University, Portland, OR, United States; ^5^ Department of Cell, Developmental & Cancer Biology, Oregon Health & Science University, Portland, OR, United States

**Keywords:** AML – acute myeloid leukemia, immune therapeutics, checkpoint inhibition therapy, RNAseq analysis, functional genomic data

## Abstract

**Introduction:**

The implementation of small-molecule and immunotherapies in acute myeloid leukemia (AML) has been challenging due to genetic and epigenetic variability amongst patients. There are many potential mechanisms by which immune cells could influence small-molecule or immunotherapy responses, yet, this area remains understudied.

**Methods:**

Here we performed cell type enrichment analysis from over 560 AML patient bone marrow and peripheral blood samples from the Beat AML dataset to describe the functional immune landscape of AML.

**Results:**

We identify multiple cell types that significantly correlate with AML clinical and genetic features, and we also observe significant correlations of immune cell proportions with *ex vivo* small-molecule and immunotherapy responses. Additionally, we generated a signature of terminally exhausted T cells (T_ex_) and identified AML with high monocytic proportions as strongly correlating with increased proportions of these immunosuppressive T cells.

**Discussion:**

Our work, which is accessible through a new “Cell Type” module in our visualization platform (Vizome; http://vizome.org/), can be leveraged to investigate potential contributions of different immune cells on many facets of the biology of AML.

## Introduction

AML is a blood cancer with an average 5-year survival of approximately 29% (1) and is characterized by an uncontrolled expansion of abnormal myeloid-lineage cells commonly referred to as “blasts”. In the United States for the year 2020, there were 60,530 new cases of leukemia of which AML encompassed roughly 1/3 (19,940), but disproportionately accounted for nearly half of the deaths (11,180 of 23,100 all leukemia) (SEER). AML is a genetically heterogeneous cancer, with the most commonly mutated genes, *FLT3, NPM1*, and *DNMT3A*, only encompassing approximately 30% of all AML patients (2). While targeted therapy discoveries have expanded greatly in the past decade, there are still many confounding variables that dampen responses in patients. Understanding these underlying variables affecting drug response is critical to achieving durable remissions by treating patients with tailored drug regimens.

As a blood cancer, AML cells are uniquely situated to interact with a plethora of immune cells either in the bone marrow or in the periphery. Numerous reports have described different mechanisms by which AML cells interact with immune cells to disrupt homeostasis via secretion of and/or increased responsiveness to pro-inflammatory cytokines (3–5), and promotion of T cell exhaustion (6–8). Furthermore, groups have begun investigating the connection between certain somatic mutations and expansion of immunosuppressive cell types, such as TP53 mutations and increased Tregs (9) or DNMT3A mutations attenuating T_H_1 macrophage polarization (10). However, these studies are limited by small patient cohorts or restricted to murine models. This prompted us to describe the functional immune landscape of AML via deconvolution of bulk RNA-seq from 560 AML patient samples and mapping to an expansive dataset of clinical annotations and *ex vivo* drug responses.

## Results

### Immune landscape of AML

**Figure 1:**
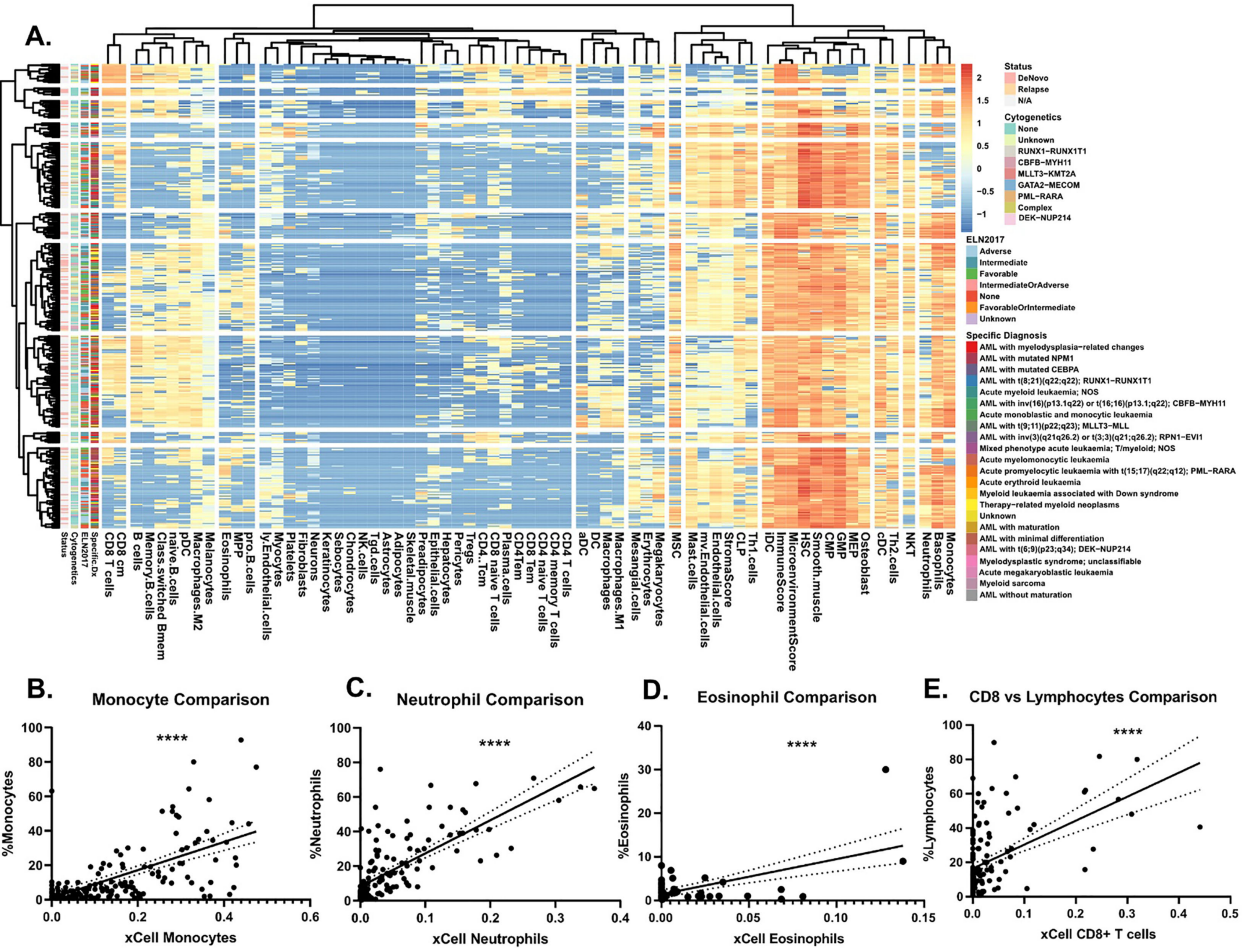
Immune landscape of AML. **(A)** Heatmap representing log2 transformed immune cell proportion estimations calculated by xCell for 252 AML patient sample peripheral blood aspirates. Cell types are grouped by Euclidean distance and patient samples by correlation. When known, relapse versus *de novo* status, ELN2017, and specific diagnosis annotations, as defined in 2, are denoted on left axis. **(B-E)** Comparison of clinical cell type proportions as determined by clinical flow cytometry, which was mined from electronic medical records of hematology/pathology clinical flow cytometry results (2, 12), at specimen acquisition versus xCell estimations for **(B)** monocytes, **(C)** neutrophils, **(D)** eosinophils, and **(E)** CD8+ T cells versus lymphocytes. Significance determined by Pearson correlation. Solid black line represents the linear line of best fit and the dotted lines on either side represent the 95% confidence interval.

**Figure 2:**
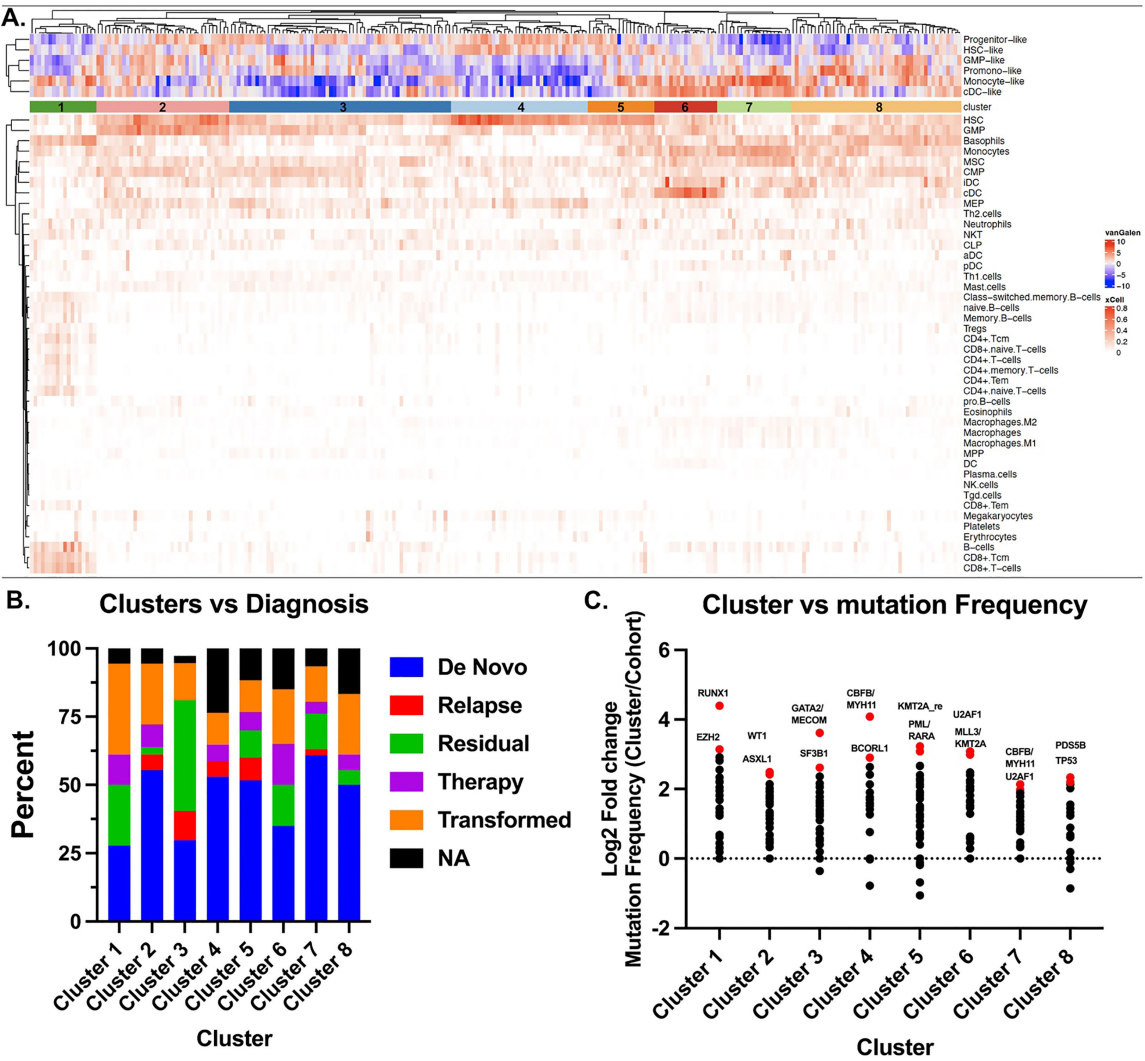
Stochastic clustering identifies correlates with diagnosis and mutational status. **(A)** xCell scores were used to generate clusters of patients using the ConsensusClusterPlus R package. Each column represents a single patient and each row corresponds to a cell type. Patients were clustered using PAM with Euclidean distance. An additional heatmap demonstrating the corresponding Van Galen cell types which were previously calculated^12^ are overlaid at the top. **(B)** Disease stage was determined from clinical annotations to annotate the 8 clusters identified in a. by Consensus Cluster. **(C)** Mutation frequencies were calculated as a proportion of the total number of patients in the respective cluster. The cohort frequency was calculated from the samples in all 8 clusters and subtracted from the individual cohort frequency to determine the change in frequency for each cluster. Data is represented Log2 transformed. Top 3-4 mutations are highlighted and color-coded red.

To assess the immune landscape of AML we scored bulk RNA-seq data to annotate proportions of various cell types using the xCell R package (11) in 560 AML patients within the Beat AML dataset (12) (**Figure 1A**, **Supplementary Figure S1A**). These cell type proportions can be explored and visualized using our Beat AML data visualization platform, Vizome (http://vizome.org/). As expected, we found bone marrow aspirate samples had decreased proportions of lymphoid populations as compared to the peripheral blood samples (**Supplementary Figures S1B–E**). We validated the accuracy of the xCell scores by comparing with flow cytometry measurements from clinical hematology/pathology testing and found strong correlation of xCell predictions with flow cytometry measurements for all cell types (**Figures 1B–E**). To investigate potential overlap with patient features, we performed hierarchical clustering of patients and cell types using the ConsensusClusterPlus R package (13) (**Figure 2A**). We evaluated the resulting clusters and found that k=8 clusters had the greatest stability (**Supplementary Figures S2A, B**). We further characterized these clusters based on their specific diagnosis and mutational status. We first annotated clusters with specific diagnoses for each specimen (**Figure 2B**). Intriguingly, Cluster 1, which had higher proportions for multiple CD4 and CD8 T cell lineages, was also enriched for transformed specimens and had the lowest proportion of *de novo* AMLs of all the clusters. Next, we assessed mutational patterns in each cluster (**Figure 2C**). We found that the T cell high cluster 1 was enriched for RUNX1 and EZH2 mutations. Conversely, Cluster 8, which had the lowest scores for T cell lineages were enriched for PDS5B and TP53 mutations. TP53 mutated AMLs have previously been reported to be associated with increased immune suppression (9, 14) and TP53 mutations are associated with a significantly worse prognosis and outcome (2, 9). Additionally, Williams et al. found that TP53 mutations were enriched in patients with higher expression of inhibitory immune checkpoint molecules on T cells in AML patients (15). Taken together, we identified clusters of AML patients who exhibit low T cell signatures that are associated with de novo TP53 mutant AMLs.

### Immune cell signatures predictive of small-molecule responses and outcome

**Figure 3:**
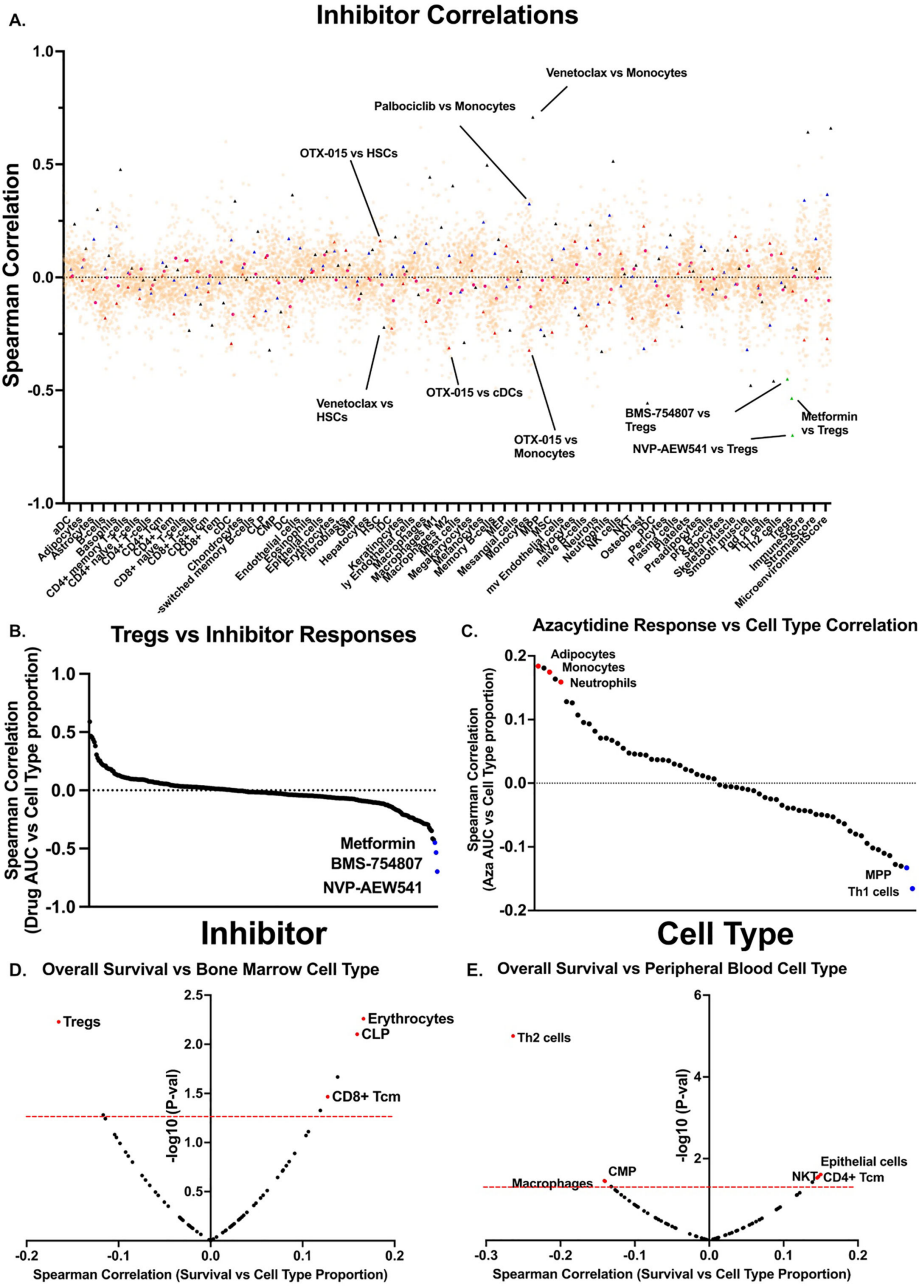
Cell type proportions correlate with small-molecule inhibitor responses in AML patient samples. **(A)** Scatter plot showing all inhibitor correlations versus all cell types. Inhibitor correlations were calculated using *ex vivo* area under the curve values derived from 2 and restricted to inhibitors with at least 20 unique patient samples. Red triangles denote BETi OTX-015 responses and highlight monocyte and cDC correlations. Black triangles denote BCL2i venetoclax and highlights monocytes and HSCs. Blue triangles denote palbociclib correlations and highlights monocytes. Green triangles denote Treg correlations and highlight IGF-1Ri NVP-AEW541, BMS-754807, and metformin. **(B)** Plot of all inhibitor AUC correlations versus. Tregs only as calculated in a. **(C)** Plot of all cell types versus HMA azacytidine, highlighting strongly positive correlations in red and negative correlations in blue, as calculated in a. **(D-E)** Spearman correlations were calculated between overall survival for corresponding patient **(D)** bone marrow and **(E)** peripheral blood samples versus. xCell cell types. Red dashed line marks significance threshold for each plot. Points of interest are highlighted in red.

We next determined cell type correlations with *ex vivo* small-molecule responses in the same patient samples. We restricted correlations to only include inhibitors with greater than 20 unique patient responses. This cutoff yielded 152 unique inhibitor monotherapies (**Figure 3A**). We and others previously described BETi and venetoclax or palbociclib as having opposing responses in monocytic AMLs, whereby BETi sensitivity is highest in monocytic AMLs (16) whereas venetoclax (17–21) and palbociclib (16) are most sensitive in undifferentiated AMLs. We were able to replicate these patterns of response as both venetoclax and palbociclib exhibited resistance in samples with high monocyte scores whereas the BETi, OTX-015, showed sensitivity in samples with high monocyte scores, thus, validating this approach. We identified many novel correlations between inhibitor responses and different cell types. Focusing on responses in samples with high proportions of Tregs, the top three inhibitors with increased effectiveness were NVPAEW-541, BMS-754807, and metformin, which target insulin signaling pathways via inhibition or downregulation of the IGF-1 receptor (**Figure 3B**). Additionally, we can focus on the responses of a single drug globally. Examination of the FDA approved hypomethylating agent (HMA), azacitidine (**Figure 3C**), reveals that resistance correlates with increased scores of adipocytes (r=0.18), monocytes (r=0.17), and neutrophils (r=0.16), whereas, Th1 cells (r=-0.13) and MPP cells (r=-0.17) correlate with azacitidine sensitivity. We next evaluated patient outcomes versus individual cell type proportions. We found that Treg signatures significantly correlate with worse survival in bone marrow aspirates, whereas, T_H_2 T cell and macrophage signatures correlate with worse survival in peripheral blood specimens (**Figures 3D, E**). Tregs have been previously shown to be correlated with a worse prognosis in AML (6, 22). In total, we describe a novel, publicly available tool with which one can utilize to interrogate inhibitor-immune cell interactions and how they may influence survival in AML.

### Monocyte high patient samples have decreased *ex vivo* responses to ICB therapy and higher Tex signatures

**Figure 4:**
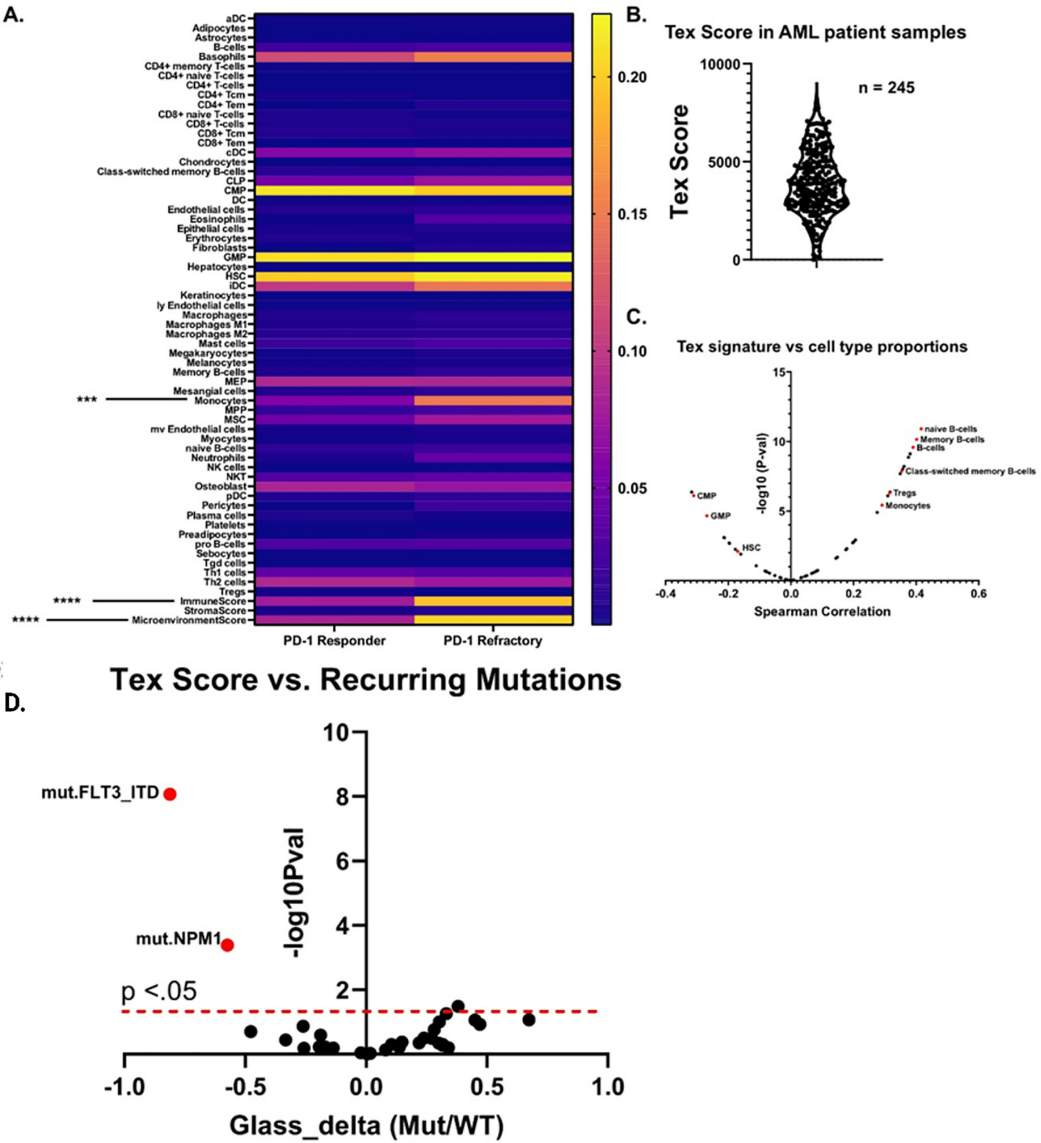
Monocytic signatures correlated with *ex vivo* resistance to immune checkpoint blockade and have increased signatures of T cell exhaustion. **(A)** Heatmap which displays the aggregate cell proportion estimate versus anti-PD1 *ex vivo* response for samples described by Lamble and Kosaka et al. 2019. Significance was calculated for all cell types via 2-way anova (n = 9 PD-1 Responder, n = 6 PD-1 refractory), ***p<.001, ****p<0.0001. **(B)** Distribution of Tex scores from peripheral blood samples within the Beat AML database. The Tex score was generated by creating a signature based on top enriched genes from previous sequencing data on exhausted T cells from (23) and (24). Higher scores correlate with increased estimated proportions of exhausted T cells. **(C)** Volcano plot showing the spearman correlations between the Tex score and all other cell types from xCell. An assortment of example cell types with either significant positive or negative enrichments are highlighted in red. **(D)** For each recurring AML mutation, we computed the difference in Tex score between mutated and wild type (shown as points). This difference is reported in terms of a standardized effect size (Glass’s delta relative to wild type; x-axis). The vertical axis indicates mutational association with increased Tex score on the right, and with decreased Tex score on the left. FDR-corrected significance of these differences is given on the y-axis with a dashed line indicating the 0.05 level.

We next asked whether any cell type signatures predicted *ex vivo* immune checkpoint response. Lamble and Kosaka et al. (8) recently evaluated the efficacy of ICB therapy in 49 bone marrow aspirate samples within the Beat AML database. Of those, 18 had dysfunctional T cells – as determined by reduced proliferative capacity and cytokine secretions. Of these samples with dysfunctional T cells, 9 were rescuable with *ex vivo* treatment with anti-PD1 blockade, and 6 were refractory. We first asked whether any xCell cell type scores significantly differed between these two groups and found that monocytes were significantly higher in anti-PD1 refractory samples (**Figure 4A**). We found this particularly interesting as Van Galen et al. (25) had previously noted that monocytic AMLs had immunosuppressive features. ICB therapy is thought to specifically re-invigorate progenitor exhausted T cells (TPEx) and retain the highest anti-tumor activity (23, 26). Conversely, exhausted CD8 T cells (Tex), which terminally differentiate from TPEx, have reduced effector function and anti-tumor activity. Additionally, patients with higher TEx/TPex ratios are resistant to ICB therapy (27, 28). Thus, given our previous data suggesting that monocytes/monocytic AMLs correlate with *ex vivo* anti-PD1 resistance and that anti-PD1 resistance has been linked to increased proportions of exhausted CD8^+^ T cells, we generated a custom T cell exhaustion signature using previously deposited sequencing data (24, 29, 30) to investigate whether this Tex signature correlates with higher proportions of monocytes or monocytic differentiation programs (**Figure 4B**). We then asked which cell types correlated with our Tex score, excluding CD8+ T cells, and indeed found that monocytes significantly positively correlated whereas undifferentiated myeloid cells such as HSCs, GMPs, and CMPS negatively correlated (**Figure 4C**). We next asked whether this Tex score predicted *ex vivo* responses to anti-PD1 therapy by correlating Tex scores with PD-1 response designations. Finally, we determined mutational correlation with TEx scores. We restricted this analysis to only include mutations which were recurring in at least 5 different specimens. This left 27 total recurring genetic mutations. For this set, we evaluated spearman correlations for all mutations and their associated Tex score. We found that FLT3-ITD and NPM1 were significantly negatively correlated with Tex scores. No mutations correlated positively with Tex scores, suggesting that the T cell exhaustion is potentially uncoupled to mutation status in AML (**Figure 4D**). However, this may also be driven by sampling errors due to the small number of mutations. In summary, we find that high monocyte scores are correlated with resistance to anti-PD1 therapy *ex vivo*.

## Discussion

The contributions of non-leukemia cells towards drug response and survival is an actively growing field but has many remaining questions. Here we survey the immune microenvironment of AML to better understand underlying mechanisms of drug – immune cell interactions and provide a tool for leukemia researchers to interrogate this axis. Utilizing *in silico* approaches we characterize the microenvironment of ~600 AML patient bone marrow and peripheral blood aspirates. We validated these approaches using available clinical flow cytometry data, where available, and found that xCell deconvolution accurately predicted cell type proportion. Our data identified TP53 and PDS5B mutated AMLs as potentially being less immunologically active with dramatically decreased proportions of naïve and effector CD8+ T cells. Additionally, we find that the T cell enriched cluster are primarily transformed AMLs, highlighting the potential importance of immune dysregulation in relapsed and transformed settings. We further correlated 152 small-molecule inhibitors with cell type proportions and identified numerous interactions. We were able to validate these approaches by investigating correlations between BETi, venetoclax, and palbociclib with monocytes, whose responses have been described by us and others as tethered to monocytic differentiation (16, 19, 20). In addition, we identified unpredicted responses of IGF1R/insulin inhibitors that are potentially tethered to Tregs, suggesting insulin growth factor receptor signaling as a potentially rational therapeutic strategy in patients with high Tregs. Studies have demonstrated that activation of IGF-1R induces Tregs (31) and that insulin resistance correlates with decreased Tregs (32). Our *in silico* data suggests that this is a targetable axis in AML patients and that its enhanced efficacy may be driven by targeting Tregs. Finally, we described the potential connection between monocytes/monocytic differentiated AMLs in predicting *ex vivo* ICB response. We show that monocyte signatures are significantly increased in AML samples with dysfunctional T cells that are refractory to ICB therapy. We further test this connection by generating a signature of exhausted T cells, which are known to drive ICB therapy resistance, and find that they indeed correlate significantly with monocyte signatures. Future studies will focus on biological validation of novel inhibitor cell type correlations, as well as further validation of associations using functional *ex vivo* immune testing data.

## Methods

### xCell enrichment scoring of RNA-seq data

Previously deposited data (2, 12) was re-processed using Kallisto v0.46.2 (33) relative to Ensembl GRCh37 v75 transcripts. Gene-level expression values were derived from summed transcript abundance. Inhibitor responses, variant calls, processed expression data, and clinical annotations are publicly available in our Vizome data visualization platform (http://vizome.org/aml2; see https://biodev.github.io/BeatAML2/ for frequently asked questions). Clinical flow calls used to compare xCell (v1.1.0) proportions were also derived from the Beat AML dataset.

### Heatmaps

Heatmaps were generated by log2 transforming cell proportion estimations for all unique AML patient sample peripheral blood aspirates (n=252). Cell types are clustered by Euclidean distance and patient samples by Pearson’s correlation. The R package “Pheatmap” was used to generate all heatmaps. Clustering of cell types versus specimen was performed using ConsensusClusterPlus (v1.54.0) using partitioning around medoids (PAM) (34) based on Euclidean distance for the inner clustering and average linkage hierarchical clustering for the outer clustering.

### Clinical flow data

Flow cytometry data was mined from electronic medical records derived from clinical hematology/pathology testing as previously described (2, 12).

### Inhibitor correlations with cell types

Inhibitor-cell type correlations were generated for all inhibitors in the Beat AML database with at least 20 unique patient samples. Details on the drug viability assay and data processing can be found in Tyner et al. 2018 (2) and Bottomly et al. 2022 (12). This resulted in 152 unique inhibitor monotherapies or combination therapies. Spearman correlations were then generated for all samples comparing inhibitor area’s under the curve (AUCs) versus xCell cell type proportions.

### Anti-PD1 therapy versus cell type proportions

*Ex vivo* aPD1 responses were determined based on Lamble and Kosaka et al. (8), which identified 6 anti-PD1 resistant and 9 anti-PD1 responding samples. Significance for all cell types between anti-PD1 refractory and responder was calculated using 2-way ANOVA and denoted on the heatmap.

### Terminally exhausted T cell score

The terminally exhausted T cell score was calculated using xCell (`rawEnrichmentAnalysis` function) with gene-sets derived from Jadhav et al. (23) and Man et al. (24) and calculated for all AML peripheral blood samples.

### Statistical testing

Unless otherwise stated, * represents P values less than.05, ** less than.01, *** less than.001, and **** less than.0001.

## Data availability statement

Publicly available datasets were analyzed in this study. These data can be found here: All raw and processed sequencing data, along with relevant clinical annotations have been submitted to dbGaP and Genomic Data Commons and are publicly available. The dbGaP study ID is 30641 and accession ID is phs001657.v2.p1 (https://www.ncbi.nlm.nih.gov/projects/gap/cgi-bin/study.cgi?study_id=phs001657.v2.p1). In addition, all data can be accessed and queried through our online, interactive user interface, Vizome, at vizome.org/aml2.

## Ethics statement

The studies involving human participants were reviewed and approved by Oregon Health & Science University Institutional Review Board. Written informed consent to participate in this study was provided by the participants’ legal guardian/next of kin.

## Author contributions

KR: formal analysis, investigation, visualization, methodology, writing–original draft, writing–review and editing. DB: formal analysis, investigation, visualization, methodology, writing-review and editing, resources, data curation. WY: methodology, writing–review and editing. NL: resources. MV: data curation. SM: formal analysis, investigation, visualization, methodology, writing-review and editing, resources, data curation. JT: Conceptualization, resources, formal analysis, supervision, funding acquisition, investigation, visualization, methodology, writing–original draft, project administration, writing–review and editing. All authors contributed to the article and approved the submitted version.
