# Supplementary material for: Immune cell proportions correlate with clinicogenomic features and ex vivo drug responses in acute myeloid leukemia
Source: Front Oncol. 2023 Jun 8;13:1192829. doi: 10.3389/fonc.2023.1192829 (PMC10285384; doi:10.3389/fonc.2023.1192829)
Supplement: Supplementary file 1 [file Image_1.pdf]

Supplemental Figure 1: Specimen type versus cell type comparisons

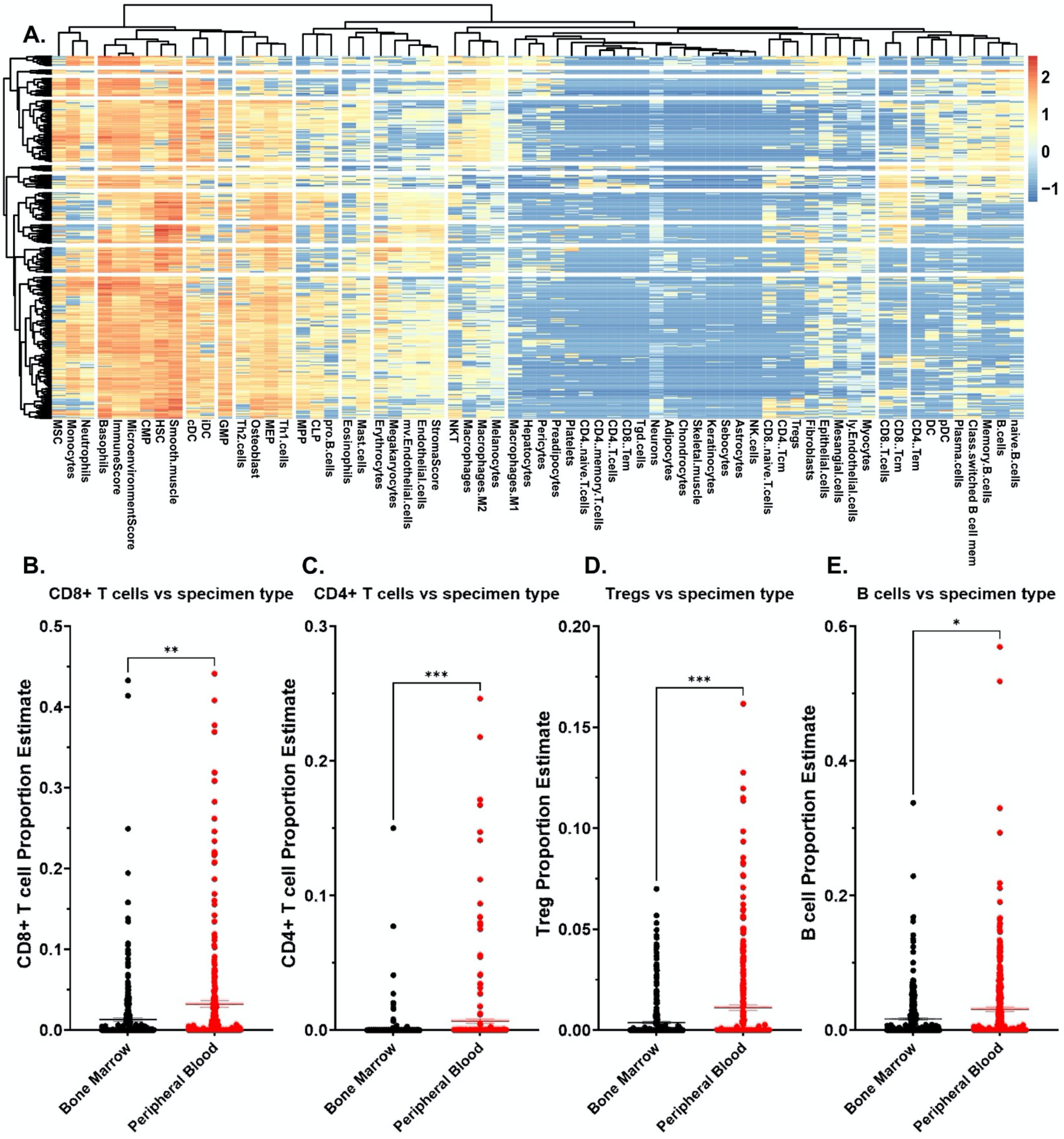

a.) Heatmap representing log2 transformed immune cell proportion estimations calculated by xCell for 362 AML patient sample bone marrow aspirates.

b-e.) Comparisons of the xCell determined cell type proportions between peripheral blood and bone marrow aspirates against b.) CD8+ T cells, c.) CD4+ T cells, d.) Tregs, and e.) B cells. Significance determined by Mann-Whitney T test.
