# Supplementary material for: Immune cell proportions correlate with clinicogenomic features and ex vivo drug responses in acute myeloid leukemia
Source: Front Oncol. 2023 Jun 8;13:1192829. doi: 10.3389/fonc.2023.1192829 (PMC10285384; doi:10.3389/fonc.2023.1192829)
Supplement: Supplementary file 2 [file Image_2.pdf]

Supplemental Figure 2: Consensus Cluster Plus CDF versus kmeans cluster number

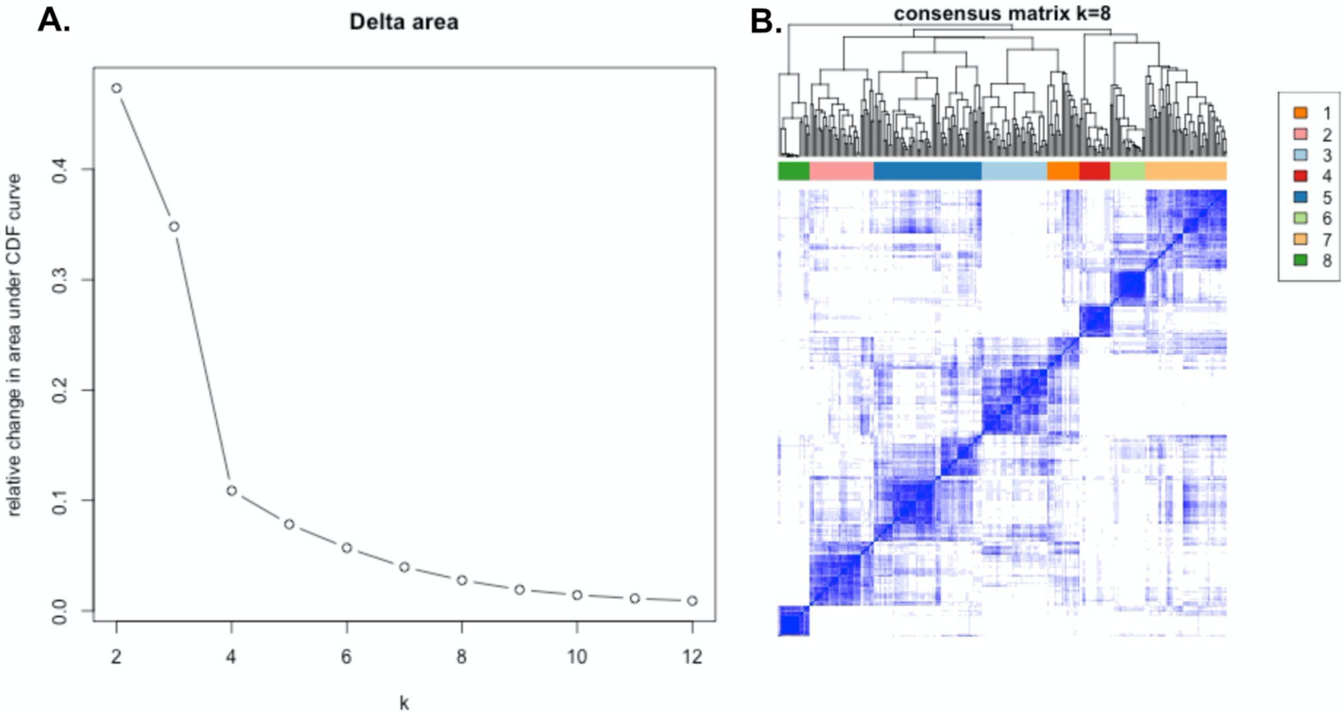

a.) Plots the relative change in the AUC of the Cumulative Distribution Function (CDF) for kmeans clusters 2-12. Plot generated using the ConsensusClusterPlus R package.

b.) A generated plot of the consensus matrix for kmeans 4 (as used in main figure 2) as determined by ConsensusClusterPlus.
